# Severe Pharyngodynia Followed by Migratory Polyarthritis and High Fever in Young Immigrants: Remember That Rheumatic Fever Is Still Relevant in 2020!

**DOI:** 10.1155/2020/8854868

**Published:** 2020-11-04

**Authors:** Kalliopi Azariadis, George Giannoulis, Stella Gabeta, Anastasia Michail, Kalliopi Zachou, George N. Dalekos

**Affiliations:** ^1^Department of Medicine and Research Laboratory of Internal Medicine, National Expertise Center of Greece in Autoimmune Liver Diseases, General University Hospital of Larissa, Larissa 41110, Greece; ^2^Institute of Internal Medicine and Hepatology, Larissa 41447, Greece

## Abstract

Acute rheumatic fever (ARF) is the immune-mediated sequelae of untreated group-A streptococcal infection. In this regard, rheumatic heart disease is the most prominent manifestation with devastating long-term complications. In the postantibiotic era, ARF is extremely rare in high-income countries; thus, its diagnosis might escape the clinicians' notice. However, its incidence remains high not only in certain low- and middle-income regions with poor public health systems but also in socioeconomically vulnerable populations residing in high-income countries. Herein, we report two cases of ARF in young immigrant adults in order to highlight the need for increased clinical suspicion to establish a prompt and timely diagnosis of ARF and describe in detail its differential diagnosis and approach to treatment.

## 1. Introduction

*Streptococcus pyogenes* (group-A *Streptococcus*, GAS) infections are still a major health problem worldwide as they can result not only in noninvasive infectious diseases such as pharyngitis (representing almost 30% of all pharyngitis cases in primary care) and impetigo but also in invasive infections, such as septic arthritis, pneumonia, scarlet fever, and necrotizing fasciitis [1]. Of interest, however, GAS can cause a variety of postinfectious, immune-mediated diseases including poststreptococcal glomerulonephritis, rheumatic heart disease (RHD) and its harbinger, acute rheumatic fever (ARF), and, maybe, the development of pediatric autoimmune neuropsychiatric disorders associated with streptococcal infections (PANDAS) [1, 2]. In this context, it is well known that ARF can occur as the immune-mediated sequelae in 3–5% of untreated GAS cases at a median of 2-3 weeks after the infection [1]. Usually, the first episodes of ARF occur just before adolescence and wane with aging, being rare in adults older than 35 years and occurring only occasionally in people older than 45 years [1].

After World War II, improvement in living conditions and medical care led to almost complete eradication of ARF in high-income countries (prevalence < 2/100,000 school-aged children) [3]. However, incidence remains high in certain socioeconomically vulnerable populations residing in high-income countries and in low- and middle-income regions, resulting in almost 40 million cases of RHD (age-standardized ratio: 500.6/100,000 population) and 285,517 deaths (age-standardized mortality: 3.7/100,000 population) worldwide in 2017 [3], even though underestimation of the true disease burden is still likely as there are many difficulties in case ascertainment [2, 4]. Indeed, previous studies in developing countries based on echocardiographic screening in children and adolescents have shown high echocardiographically confirmed prevalence of RHD suggesting that many cases of ARF were subclinical [5, 6].

Clinical manifestations of ARF include fever, carditis (mitral and/or aortic valve regurgitation), polyarthritis, Sydenham's chorea (though it is presented later, up to 6 months after GAS infection), subcutaneous nodules, and erythema marginatum [1, 2, 7]. In the laboratory work-up, increased erythrocyte sedimentation rate (ESR) and C-reactive protein (CRP) are typical, while throat cultures are negative for GAS, and evidence for antecedent streptococcal infection could be confirmed by high or increasing titers of antibodies to streptolysin-O antigens (ASTO). The aforementioned manifestations have been incorporated in the revised Jones criteria (RJC; Table 1), which are widely used to establish a firm ARF diagnosis [8]. Apart from erythema marginatum and subcutaneous nodules, which are considered disease-specific but seen in less than 10% of cases, no other pathognomonic findings establish the diagnosis of ARF, and therefore, its differential diagnosis is sometimes difficult and should be extensive [1, 7]. In this regard, especially in low-incidence populations, ARF might escape clinical suspicion.

Herein, we present two cases of ARF in young immigrants and highlight the crucial steps in establishing the diagnosis in conjunction with approach to treatment, aiming to increase the clinical awareness of this important disease.

## 2. Case Presentation

The first patient was a 19-year-old male who was admitted to our department because of high fever (39°C) and migratory polyarthritis involving the knees, ankles, and right elbow dating back for five days. Of note, the patient reported a severe sore throat episode accompanied by pharyngodynia and odynophagia three weeks ago lasting for two weeks. He was an immigrant from Pakistan residing the last three years in Greece, in an overcrowded apartment together with 18 people of the same nationality, and is currently employed as a low-income field worker. His past medical history was unremarkable, while he denied ever having a history of current or past unsafe use of alcohol, herbal or supplements, and nasal or intravenous drug abuse. On clinical examination, the right knee and both ankles were warm, swollen with movement restriction, while a large pustule was noticed in the outer surface of the right ankle. Heart auscultation revealed a systolic murmur (1 out of 6, Levine scale) in the cardiac apex.

The second patient was an 18-year-old male, who, actually, was a roommate to the first patient, and admitted because of fever (38°C), pharyngodynia, and arthritis of the ankles during the last five days. The patient was also of Pakistani descent, immigrant to Greece for the last six months, and currently is occupied as a field worker. His symptoms began three weeks after those of the first patient. He reported never receiving medical care since early childhood, while he denied any current or past alcohol abuse, herbal or supplements, and nasal or intravenous drug use. Clinical examination revealed pharyngitis, warm, swollen, and painful ankle joints, hepatomegaly (2 cm below the right subcostal margin), and a loud precordial systolic and diastolic murmur (3 out of 6, Levine scale).

**Figure 1:**
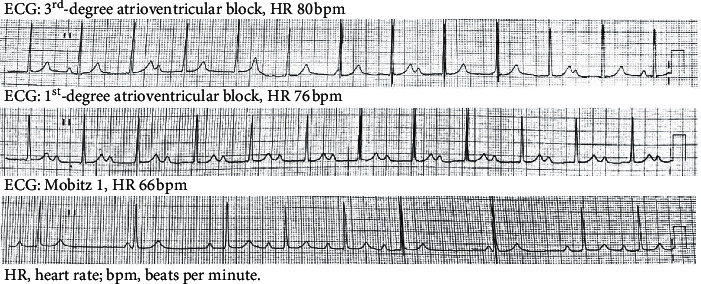
Electrocardiogram (ECG) findings in patient 2.

Laboratory work-up on admission revealed high serum levels of acute phase reactants, anemia, polyclonal hyperglobulinemia, and mild transaminasemia in both patients, while in the second case, additional cholestasis and hypoalbuminemia were recorded (Table 2). The remaining hematological, microbiological, and biochemical parameters including blood, stools, and urine cultures, as well as autoantibodies related to autoimmune rheumatic diseases including antinuclear antibodies, antineutrophil cytoplasmic antibodies, rheumatoid factors, antibodies against cyclic citrullinated peptides, and ferritin levels, were within normal limits in both patients. Multiple stool samples were also negative for ova and parasites such as *Enterobius vermicularis*, *Entamoeba histolytica*, *Cryptosporidium*, and *Giardia* species. Examination of the ankle joint synovial fluid showed increased neutrophil counts (patient 1: 21,600 neutrophils; patient 2: 24,000 neutrophils). X-rays of the chest and affected joints were unremarkable. Of note, patient 2 had an abnormal electrocardiogram (ECG) with third-degree atrioventricular block that, during hospitalization, interchanged with first-degree block and Mobitz I (Figure 1), even though he was completely asymptomatic.

Accordingly, the differential diagnosis was built in three major pillars. The first included infectious diseases manifesting with arthritis according to our local endemicity [9–15], the young age, and the epidemiological background of the patients. Actually, a serological work-up for zoonosis (*Brucella*, *Coxiella*, *Rickettsia*, *Borrelia*, *Leptospira*, *Leishmania*, and other parasites including investigation for *Trichinella*, *Entamoeba*, and *Echinococcus*), sexually transmitted diseases (syphilis, *N. gonorrhoeae*, *U. urealyticum*, *M. hominis*, and *C. trachomatis*), viral hepatitis A, B, C, and E, and human immunodeficiency virus tested negative. Tropheryma Whipple infection (Whipple's disease), which can be presented with arthritis that predates gastrointestinal symptoms, was also excluded by a polymerase chain reaction (PCR) assay performed in the synovial fluid. Tuberculosis was also ruled out based on a negative chest X-ray, negative tuberculin test, and negative synovial fluid microscopy and culture. Furthermore, the presence of aseptic arthritis was confirmed on the basis of a negative 16sRNA PCR test as we described previously [16–19] along with sterile synovial fluid cultures.

The second pillar included diverse autoimmune rheumatic diseases such as rheumatoid arthritis, systemic lupus erythematosus, psoriatic arthritis, and ankylosing spondylitis which were excluded on the absence of several clinical and laboratory criteria. The third clinical entity which was considered highly likely in both cases was ARF with or without accompanied infectious endocarditis. In the first patient, infectious endocarditis was excluded based on repeatedly negative blood cultures and transesophageal echocardiogram (TOE) confirming the absence of structural valve disorders. The second patient had established valvular heart disease and possible endocarditis since TOE revealed bicuspid aortic valve with regurgitation (3^+^/4) and stenosis (peak gradient 67.2 mmHg) with possible presence of vegetation, mitral valve regurgitation (2^+^/4), and moderate stenosis along with moderately dilated left ventricle. However, multiple blood cultures were sterile though the safer way to exclude infectious endocarditis in patient 2 would be surgical excision and valve tissue culture. As expected, throat cultures and rapid antigen tests for GAS were negative in both patients. However, GAS was isolated in the pustular fluid culture from patient 1, while ASTO titers were elevated in both patients (Table 2). The diagnosis of ARF in the first patient was based on the presence of one major (polyarthritis) and two minor (fever ≥ 38.5°C, ESR ≥ 60 mm, and CRP ≥ 3 mg/dL) RJC while in the second patient was based on the presence of established RHD and two major (carditis and oligoarthritis) and two minor RJC (fever ≥ 38.5°C, ESR ≥ 60 mm, and CRP ≥ 3 mg/dL).

With respect to treatment, patient 1 received intravenous vancomycin (15 mg/kg/bd) and ceftriaxone (2 g bid) till culture reports and oral indomethacin for 14 days for pain relief. He was discharged in good health with recommendation to receive benzathine penicillin G (1.200.000 IU) intramuscularly every twenty-eight days for at least 5 years in an attempt to prevent recurrent ARF episodes. Patient 2 received oral indomethacin for 14 days for pain relief along with intravenous ceftriaxone (2 g bid) and vancomycin (15 mg/kg/bd) for six weeks as endocarditis could not be safely excluded at the clinical ground. Subsequently, the patient submitted to aortic valve replacement with a mechanical valve. The presence of vegetation was not confirmed during surgery, and the tissue culture was sterile. The patient was discharged in good health with recommendation to receive lifelong chemoprophylaxis with azithromycin (250 mg qd) in an attempt to prevent recurrent ARF episodes. After one-year of follow-up, both patients are still in good health afebrile, without joint involvement or new ARF episode, while the laboratory markers are steadily normal. Both patients gave written informed consent for potential publication at the time of discharge.

## 3. Discussion

We report two cases of ARF in young adult immigrants, who were roommates and got sick three weeks apart from each other. The first patient had probably his first ARF attack as there was no compatible history or evidence of RHD, while the second had already established RHD. Because of the use of antibiotics and the improvement of living and sanitation conditions, ARF is nowadays quite rare in developed countries, although outbreaks may still occur. In contrast, in several developing countries, ARF and RHD continue to be a considerable public health problem [1–3, 7, 8]. Indeed, ARF is a disease emerging from poverty, overcrowding, and low standards of living conditions. As a result, these high-risk populations may be exposed repeatedly to GAS infections leading to recurrent or extended episodes of ARF increasing, in parallel, the risk of RHD development. Antibiotics in acute GAS pharyngitis shorten disease duration by only 1-2 days, but their main role is to prevent ARF and control the spread of GAS in close communities. Our cases underline that the rarity of a disease is always contextual to the demographic and epidemiological background of each patient, and therefore, clinicians should keep in mind ARF as a potential diagnosis even in 2020, when other conditions have been appropriately excluded and the demographic, clinical, and laboratory characteristics are supportive of ARF.

RHD is the most prominent complication of ARF that carries an important health burden worldwide [1–3]. Carditis is more prevalent in young adults reflecting the cumulative damage of recurrent ARF episodes in childhood and adolescence. As for other immune-mediated or autoimmune diseases that have been linked to antecedent infections, molecular mimicry (mainly between the GAS surface M-protein and host cardiac myosin) and epitope spreading are the most likely possible pathogenetic mechanisms for the development of poststreptococcal cardiac disease [2, 7, 20–25]. Of interest, in keeping with autoimmune diseases but in contrast with our cases, RHD is observed more frequently in women than men [4, 7].

Treatment of ARF is symptomatic, but unfortunately, this management does not prevent following valvular damage. Penicillin or other related antibiotics are usually administered to ARF patients in an attempt to eradicate any remaining GAS infection, but the backbone of therapy still remains the management of inflammatory response with high dose of aspirin or nonsteroidal anti-inflammatory drugs. Regarding secondary prophylaxis with antibiotics, it is clear that the risk of RHD development in patients with an ARF history is reduced [26]. Indeed, after the initial episode of ARF, long-standing antibiotic chemoprophylaxis with intramuscular injections of benzathine penicillin every 3-4 weeks seems crucial to prevent recurrent episodes and progression to RHD, allowing disease resolution in mild RHD cases [26]. However, the implementation of long-term secondary chemoprophylaxis in poor, socially close, and vulnerable communities such as immigrants and minorities is questionable [27]. These social groups are facing significant barriers in access to health services. In fact, migrants and refugees frequently have legal and administrative difficulties in acquiring a residence permit for the eligibility identification card, financial difficulties with payments for health-care services, inadequate information on access to services, language difficulties to communicate with health-care professionals, and fear towards the operation of the public services [27]. As the molecular diversity of “rheumatogenic” GAS strains has hampered effective vaccine development [28], the reduction of ARF and RHD burden is mainly based on primary prevention, and therefore, primary care physicians should be aware of this in order to uncover and diagnose promptly GAS infections, in particular, in populations with poor approach to public health systems.

In conclusion, our cases highlight the importance for physicians and especially for young internists or general practitioners to achieve a timely and prompt ARF diagnosis because even though it is an infrequent complication of GAS infections in high-resource societies, its incidence can be high among specific socioeconomically vulnerable populations, such as immigrants and refugees. ARF diagnosis requires appropriate exclusion of a wide range of diseases, but a timely diagnosis is vital as proper chemoprophylaxis can prevent the devastating complication of RHD.

## Figures and Tables

**Table 1:** Revised Jones criteria (2015). Diagnosis of acute rheumatic fever (ARF) requires two major criteria or one major and two minor criteria. Recurrent ARF episodes in patients with rheumatic heart disease (RHD) can be diagnosed even in the presence of only three minor criteria.

Major criteria	Carditis
	Polyarthritis^*∗*^
	Chorea
	Erythema marginatum
	Subcutaneous nodules

Minor criteria	Fever ≥ 38.5°C
	Erythrocyte sedimentation rate (ESR) ≥ 60 mm and/or C-reactive protein (CRP) ≥ 3.0 mg/dL^*∗*^
	Prolonged PR interval ^*∗∗*^

^*∗*^Slight modifications are applied to moderate- and high-risk populations (mono- or oligoarthritis as a major criterion, ESR ≥ 30 mm). ^*∗∗*^Unless carditis is used as a major criterion.

**Table 2:** Laboratory findings of patients 1 and 2 on admission.

	Patient 1	Patient 2	Reference values
WBC (10^3^/*μ*L)	13.4	16.3	4.5–10.5
Neutrophils (10^3^/*μ*L)	9.8	13.2	1.5–6.5
Lymphocytes (10^3^/*μ*L)	2.1	1.8	1.2–3.8
Monocytes (10^3^/*μ*L)	1.1	0.9	0.2–1
Eosinophils (10^3^/*μ*L)	0	0.03	0–0.7
Basophils (10^3^/*μ*L)	0.3	0.2	0–0.2
Ht (%)	33.6	34.9	39–46
Hb (g/dL)	10.2	11.5	13–16
PLT (10^3^/*μ*L)	354	446	140–440
ESR (mm)	102	95	2–15
INR	1.5	1.2	0.85–1.15
CRP (mg/dL)	18.3	23	0–0.7
Urea (mg/dL)	37	35	<43
Creatinine (mg/dL)	0.7	0.84	0.7–1.2
Na^+^/K^+^ (mmol/L)	131/4.1	133/4.7	136–146/3.5–5.3
Total protein (g/dL)	7.9	7.2	6.4–8.3
Albumin (g/dL)	4.3	3.4	3.5–5.2
Bilirubin (mg/dL)	1.3	0.85	<1.1
AST/ALT (IU/L)	46/43	49/44	<40
ALP/*γ*-GT (IU/L)	78/47	160/167	<120/<38
Serum protein electrophoresis	Polyclonal increase of *γ*-globulins	Polyclonal increase of *γ*-globulins	
ASTO (IU/mL)	834	1120	<200

WBC = white blood cells; Ht = hematocrit; Hb = hemoglobin; PLT = platelets; ESR = erythrocyte sedimentation rate; INR = international normalized ratio; CRP = C-reactive protein; AST = aspartate aminotransferase; ALT = alanine aminotransferase; ALP = alkaline phosphatase; *γ*-GT = gamma-glutamyl transferase; ASTO = antibodies to streptolysin-O antigens.

## Data Availability

The data used to support the findings of this study are included within the article.
